# Blowup Phenomenon of Solutions for the IBVP of the Compressible Euler Equations in Spherical Symmetry

**DOI:** 10.1155/2016/3781760

**Published:** 2016-02-03

**Authors:** Ka Luen Cheung, Sen Wong

**Affiliations:** Department of Mathematics and Information Technology, The Hong Kong Institute of Education, 10 Lo Ping Road, Tai Po, New Territories, Hong Kong

## Abstract

The blowup phenomenon of solutions is investigated for the initial-boundary value problem (IBVP) of the *N*-dimensional Euler equations with spherical symmetry. We first show that there are only trivial solutions when the velocity is of the form *c*(*t*)|**x**|^*α*−1^ **x** + *b*(*t*)(**x**/|**x**|) for any value of *α* ≠ 1 or any positive integer *N* ≠ 1. Then, we show that blowup phenomenon occurs when *α* = *N* = 1 and $c^{2}(0)+\dot{c}(0)<0$. As a corollary, the blowup properties of solutions with velocity of the form $\left(\dot{a}\left(t\right)/a\left(t\right))x+b(t)(x/\left|x\right|\right)$ are obtained. Our analysis includes both the isentropic case (*γ* > 1) and the isothermal case (*γ* = 1).

## 1. Introduction and Main Results

In this paper, we consider the *N*-dimensional Euler equations for compressible fluid:

(1) ρt+∇·ρu=0,ρut+u·∇u+∇p=0,p=Kργ,γ≥1

with boundary condition

(2) $$\begin{array}{c} {\left.\;u\cdot n\;\right|}_{\left|x\right|=1}=0, \end{array}$$

where *ρ*, **u**, and *p* represent the density, velocity, and pressure of the fluid, respectively. **n** is the unit normal vector on the unit sphere. The *γ*-law for *p* is given by (1)_3_. The fluid is called isentropic if *γ* > 1 and is called isothermal if *γ* = 1.

Euler equation (1) is one of the most important fundamental equations in inviscid fluid dynamics. Many interesting fluid dynamic phenomena can be described by system (1) [1, 2]. The Euler equations are also the special case of the noted Navier-Stokes equations, whose problem of whether there is a formation of singularity is still open and long-standing. Thus, the singularity formation in fluid mechanics has been attracting the attention of a number of researchers [3–11].

In particular, in [3, 4], the authors obtain blowup results for the IBVP of the Euler equations, namely, system (1) with boundary condition (2). By making use of the finite propagation speed property [5, 6], they are able to apply the integration method to derive differential inequalities and show that if the initial weighted functionals of velocity or momentum are large enough, then blowup occurs.

In [10], the authors consider the solutions of (1) with velocity of the form

(3) $$\begin{array}{c} u\left(\;t,x\;\right)=c\left(\;t\;\right)x \end{array}$$

and show that, by using the standard argument of phase diagram, the solutions will be expanding if *c*(0) and $\dot{c}(0)$ satisfy some inequalities. It is natural to consider the more general velocity form:

(4) $$\begin{array}{c} u\left(\;t,x\;\right)=c\left(\;t\;\right)x+b\left(\;t\;\right)\frac{x}{\left|x\right|} \end{array}$$

for the IBVP of system (1) in spherical symmetry, where *b*(*t*) is a time-dependent drifting function.

For solutions in spherical symmetry, namely, *ρ*(*t*, *x*) = *ρ*(*t*, *r*) and **u**(*t*, *x*) = *u*(*t*, *r*)(**x**/*r*), system (1) together with (2) is transformed to

(5) ρt+uρr+ρur+N−1rρu=0,t>0,  r>1,ρut+uur+pr=0,t>0,  r>1,p=Kργ,γ≥1,ut,1=0,

where *r* = |**x**| is the length of the spatial variable **x**.

Our main contributions in this paper are stated as follows.

**Theorem 1 .**

There are only trivial solutions to the *N*-dimensional Euler system (5) of the form *u* = *c*(*t*)*r* ^*α*^ + *b*(*t*) for any real *α* and integer *N* in the isentropic and isothermal cases except the case *γ* = *α* = *N* = 1. For *γ* = *α* = *N* = 1, one has the following two cases.

(1) If $c^{2}(0)+\dot{c}(0)>0$, then, for any *t*,$\;\;c^{2}(t)+\dot{c}(t)>0$, and *c*(*t*) → 0, (*ρ*, *u*)→(0,0) as *t* → *∞*.
(2) If $c^{2}(0)+\dot{c}(0)<0$, then, for any *t*,$\;\;c^{2}(t)+\dot{c}(t)<0$. Moreover, in the region where $c^{2}(t)+\dot{c}(t)<0$, *c*(*t*)→−*∞* and (*ρ*, *u*)→(*∞*, −*∞*) as *t* → *∞*.

As a corollary, we also obtain the following.

**Corollary 2 .**

Let (*ρ*, *u*) be a solution for (5) with $u=(\dot{a}\left(t\right)/a\left(t\right))r+b(t)$, *a*_0_≔*a*(0) > 0, and *γ* = *N* = 1. Then *a*(*t*) satisfies

(6) $$\begin{array}{c} \ddot{a}=\frac{\lambda }{a} \end{array}$$

for some constant *λ* ∈ *ℝ*. Furthermore, one has the following five cases.

(1) If *λ* < 0, then the solution (*ρ*, *u*)→(*∞*, −*∞*) as *t* → *T* ^*∗*^ for some finite *T* ^*∗*^ > 0.
(2) If *λ* > 0, then *ρ* is bounded above and the solution (*ρ*, *u*)→(0,0) as *t* → *∞*.
(3) If *λ* = 0 and *a*_1_ > 0, *ρ* is bounded above and the solution (*ρ*, *u*)→(0,0) as *t* → *∞*.
(4) If *λ* = 0 and *a*_1_ = 0, then the solution is trivial.
(5) If *λ* = 0 and *a*_1_ < 0, then the solution (*ρ*, *u*)→(*∞*, −*∞*) as *t* → −*a*_0_/*a*_1_.

## 2. Lemmas

It is well-known that *ρ* is always positive if the initial datum *ρ*(0, *r*) is set to be positive. Thus, we suppose *ρ*(0, *r*) > 0 in the following to avoid the trivial solutions *ρ* ≡ 0.

**Lemma 3 .**

For *γ* > 1, one has

(7) $$\begin{array}{c} {\left[\;\frac{\rho^{\gamma -1}}{\gamma -1}\;\right]}_{t}+{\left[\;\frac{\rho^{\gamma -1}}{\gamma -1}\;\right]}_{r}u+\rho^{\gamma -1}\left[\;u_{r}+\frac{N-1}{r}u\;\right]=0. \end{array}$$

**Proof**

From (5)_1_, one has

(8) $$\begin{array}{c} \rho_{t}+\rho_{r}u+\rho \left(\;u_{r}+\frac{N-1}{r}u\;\right)=0. \end{array}$$

Multiply both sides by *ρ* ^*γ*−2^. Then, the result follows.

**Lemma 4 .**

For *γ* > 1, one has

(9) ργ−1γ−1r=−1Kγut+uur,

(10) ργ−1=ργ−1t,1−γ−1Kγ∫1rut+uurt,sds,

(11) ργ−1γ−1t=ddtργ−1t,1γ−1−1Kγ∫1rut+uurtt,sds.

**Proof**

From (5)_2_, one has

(12) ut+uur+Kργ−2ρr=0,ut+uur+Kργ−1γ−1r=0

and the results follow.

Similarly, we have the following two lemmas for *γ* = 1.

**Lemma 5 .**

For *γ* = 1, one has

(13) $$\begin{array}{c} {\left[\;\ln\rho \;\right]}_{t}+{\left[\;\ln\rho \;\right]}_{r}u+\left(\;u_{r}+\frac{N-1}{r}u\;\right)=0. \end{array}$$

**Lemma 6 .**

For *γ* = 1, one has

(14) ln⁡ρr=−1Kur+uur,

(15) ln⁡ρ=ln⁡ρt,1−1K∫1rur+uurt,sds,

(16) ln⁡ρt=ddtln⁡ρt,1−1K∫1rur+uurtt,sds.

Lastly, one has the following lemma that will be used to prove that there are only trivial solutions when *u*(*t*, *r*) = *c*(*t*)*r* + *b*(*t*) and *γ* > 1.

**Lemma 7 .**

Consider the following dynamical system

(17) c3+A1cc˙+B1c¨=0,c3+A2cc˙+B2c¨=0

with *A*_1_ ≠ *A*_2_ or *B*_1_ ≠ *B*_2_. If *A* ≠ 0, *B* ≠ 0, and

(18) $$\begin{array}{c} A\left(\;A_{1}B_{2}-A_{2}B_{1}\;\right)=2B^{2}, \end{array}$$

then (17) is equivalent to

(19) $$\begin{array}{c} \frac{A}{2}c^{2}+B\dot{c}=0, \end{array}$$

where

(20) A≔A2−A1,B≔B2−B1.

Otherwise, the solution to (17) is trivial.

**Proof**

If *A*_1_ *B*_2_ − *A*_2_ *B*_1_ = 0, then it is clear that *c* = 0 is the only solution. So we suppose *A*_1_ *B*_2_ − *A*_2_ *B*_1_ ≠ 0. One has from (17) that

(21) $$\begin{array}{c} Ac\dot{c}+B\ddot{c}=0. \end{array}$$

If *B* = 0 and *A* ≠ 0, then *c* = 0 is the only solution.

If *A* = 0 and *B* ≠ 0, then *c* = 0 is the only solution.

So, we suppose both *A* and *B* are not zero.

From (21), one has

(22) $$\begin{array}{c} \frac{A}{2}c^{2}+B\dot{c}=\xi , \end{array}$$

for some constant *ξ*.

From (21) and (22), one has

(23) c¨=−ABcc˙,c˙=1Bξ−A2c2.

Thus, from (17)_1_, one has

(24) c1−AA1B2−A2B12B2c2+A1B2−A2B1B2ξ=0.

If 1 − *A*(*A*_1_ *B*_2_ − *A*_2_ *B*_1_)/2*B* ^2^ ≠ 0, then *c* = 0 is the only solution. So we suppose it is zero; that is, (18) holds. Then, if *ξ* ≠ 0, *c* = 0 is the only solution. So we suppose *ξ* = 0. Thus, we have

(25) $$\begin{array}{c} \frac{A}{2}c^{2}+B\dot{c}=0. \end{array}$$

Conversely, if one has (18) and (19), then system (17) is satisfied. The proof is complete.

## 3. Proofs of Main Results

**Proposition 8 .**

Assume *γ* > 1. Then there are only trivial solutions to the *N*-dimensional Euler system (5) of the form *u* = *c*(*t*)*r* ^*α*^ + *b*(*t*) with *α* ≠ 1.

**Proof**

First, we set

(26) $$\begin{array}{c} u\left(\;t,r\;\right)=c\left(\;t\;\right)r^{\alpha }+b\left(\;t\;\right). \end{array}$$

From (5)_4_, we have

(27) $$\begin{array}{c} u=c\left(\;t\;\right)\left[\;r^{\alpha }-1\;\right]. \end{array}$$

Then,

(28) ut+uur=c˙rα+αc2r2α−1−αc2rα−1−c˙,

(29) ut+uurt=c¨rα+2αcc˙r2α−1−2αcc˙rα−1−c¨,

(30) ur+N−1ru=α+N−1crα−1−N−1cr−1.

For *N* > 1, if *α* = 0, then *u* = 0 from (27). It follows from (7) and (9) that *ρ*(*t*, *r*) is independent of *t* and *r*, respectively. Thus, *ρ* is a constant.

For *α* ≠ 0 and −1, after substituting (28), (29), and (30) into (9), (11), and (7), respectively, we see that (7) becomes

(31) D1r3α−1+D2r2α+D3r2α−1+D4rα+1+D5rα+D6rα−1+D7r1+D8r0+D9r−1=0

for all *r* ≥ 1, where *D*_*k*_ are functions of *t* only. More precisely, one has

(32) D1=−2α+γ−1α+N−12Kγc3,D2=−γ−1α+N−1+2α+2Kγα+1cc˙,D3=γ−12α+3N−3+4α2Kγc3,D4=−1Kγα+1c¨,D5=γ−1α+N+1+γ−1N−1α+1+4·1Kγcc˙,D6=−α+γ−1N−1Kγc3+α+N−1Fc,D7=1Kγc¨,D9=−N−1Fc,

where

(33) $$\begin{array}{c} F≔\rho^{\gamma -1}\left(\;t,1\;\right)-\frac{\gamma -1}{K\gamma }\left[\;\frac{\alpha }{\alpha +1}\dot{c}+\frac{c^{2}}{2}\;\right]. \end{array}$$

Note that we omitted *D*_8_ as it is irrelevant in the proof.

If *α* ∉ {0,1/3,1/2,2/3,1, 2}, then the powers 3*α* − 1 and 2*α* − 1 are different and unique among the powers in (31). In this case, one has

(34) D1=0,D3=0.

As the two constants 2*α* + (*γ* − 1)(*α* + *N* − 1) and (*γ* − 1)(2*α* + 3*N* − 3) + 4*α* cannot be both zero for *N* ≠ 1, we conclude that *c* = 0. Hence, *u* = 0 and *ρ* is a constant.

For *α* ∈ {1/3,1/2,2/3,2}, we have Table 1.

For *α* = 1/3 or 2/3, as 2*α* − 1 is unique among other powers, one has

(35) $$\begin{array}{c} D_{3}=\frac{\left(\gamma -1\right)\left(2\alpha +3N-3\right)+4\alpha }{2K\gamma }c^{3}=0. \end{array}$$

As (*γ* − 1)(2*α* + 3*N* − 3) + 4*α* ≠ 0, we conclude that *c* = 0. Thus, *u* = 0 and *ρ* is a constant.

For *α* = 1/2, as *α* − 1 and −1 are different and unique among other powers, one has

(36) D6=0,D9=0,

which is reduced to

(37) $$\begin{array}{c} -\frac{\alpha +\left(\gamma -1\right)\left(N-1\right)}{K\gamma }c^{3}=0 \end{array}$$

for *N* ≠ 1. As *α* + (*γ* − 1)(*N* − 1) ≠ 0, we conclude that *c* = 0. Thus, *u* = 0 and *ρ* is a constant.

For *α* = 2, as 3*α* − 1 is unique among other powers, one has

(38) $$\begin{array}{c} D_{1}=-\frac{2\alpha +\left(\gamma -1\right)\left(\alpha +N-1\right)}{2K\gamma }c^{3}=0. \end{array}$$

As 2*α* + (*γ* − 1)(*α* + *N* − 1) ≠ 0, we conclude that *c* = 0. Thus, *u* = 0 and *ρ* is a constant.

Next, we consider the case *α* = −1.

For *α* = −1, the corresponding equation of (31) is

(39) E1ln⁡r+E2r−1ln⁡r+E3r−2ln⁡r+E4r+E5r0+E6r−1+E7r−2+E8r−3+E9r−4=0

for all *r* ≥ 1, where *E*_*k*_ are functions of *t* only. As ln⁡*r* is not a rational function, one has that all *E*_*k*_ = 0. In particular, one has

(40) E8=−4+γ−13N−52Kγc3=0,E9=2−N−2γ−12Kγc3=0.

As −4 + (*γ* − 1)(3*N* − 5) and 2 − (*N* − 2)(*γ* − 1) cannot be both zero for *N* ≠ 1, we conclude that *c* = 0 and the solutions are trivial.

For *N* = 1 and *α* ≠ 1, one can show that there are only trivial solutions with similar procedures. The proof is complete.

Using similar analysis as that given for the case *γ* > 1 in Proposition 8, we obtain the following proposition for the case *γ* = 1.

**Proposition 9 .**

Assume *γ* = 1. Then there are only trivial solutions to the *N*-dimensional Euler system (5) of the form *u* = *c*(*t*)*r* ^*α*^ + *b*(*t*) with *α* ≠ 1.

Next, the crucial case *α* = 1 will be analyzed as follows.

**Proposition 10 .**

Assume *γ* > 1. Then there are only trivial solutions to the *N*-dimensional Euler system (5) of the form *u* = *c*(*t*)*r* ^*α*^ + *b*(*t*) with *α* = 1.

**Proof**

For *γ* > 1 and *α* = 1, one has

(41) D1+D2+D4=0,D3+D5+D7=0,D6+D8=0,D9=0.

(41)_1_ and (41)_2_ are equivalent to

(42) c3+A1cc˙+B1c¨=0,c3+A2cc˙+B2c¨=0,

where

(43) A1=γ−1N+4γ−1N+2,B1=1γ−1N+2,A2=3γ−1N+1+8γ−13N−1+4,B2=2γ−13N−1+4.

Note that *B*_1_ = *B*_2_ is equivalent to *N* = 1 and *A*_1_ = *A*_2_ is equivalent to

(44) $$\begin{array}{c} \gamma -1=\frac{N-5}{2N},\;N\geq 6. \end{array}$$

Thus, we have either *A*_1_ ≠ *A*_2_ or *B*_1_ ≠ *B*_2_. Moreover, condition (18) is equivalent to

(45) $$\begin{array}{c} \gamma -1=-\frac{8}{N\left(N+3\right)}, \end{array}$$

which is impossible for *γ* > 1. Thus, we conclude by Lemma 7 that there are only trivial solutions.

Next, we consider the remaining case *γ* = *α* = 1.

**Proposition 11 .**

Assume *γ* = 1. Then there are only trivial solutions to the *N*-dimensional Euler system (5) of the form *u* = *c*(*t*)*r* ^*α*^ + *b*(*t*) with *α* = 1 and *N* > 1. For *N* = *α* = 1, one has the following two cases.

(1) If $c^{2}(0)+\dot{c}(0)>0$, then, for any *t*, $c^{2}(t)+\dot{c}(t)>0$, and *c*(*t*) → 0, (*ρ*, *u*)→(0,0) as *t* → *∞*.
(2) If $c^{2}(0)+\dot{c}(0)<0$, then, for any *t*, $c^{2}(t)+\dot{c}(t)<0$. Moreover, in the region where $c^{2}(t)+\dot{c}(t)<0$, *c*(*t*)→−*∞* and (*ρ*, *u*)→(*∞*, −*∞*) as *t* → *∞*.

**Proof**

For *γ* = *α* = 1, the corresponding system of (41) is

(46) G1+G2+G4=0,G3+G5+G7=0,G6+G8=0,G9=0,

where
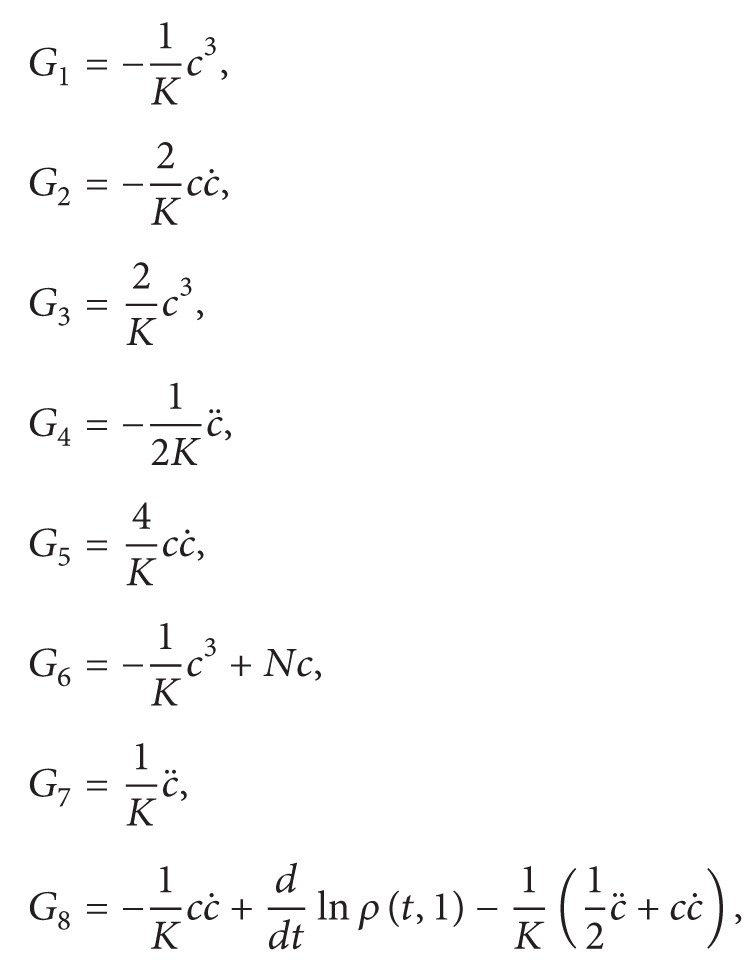
(47)

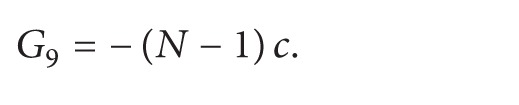
(48) It is clear that from (46)_4_ and (48) we have only the trivial solutions if *N* ≠ 1. So we suppose *N* = 1. Then (46) is equivalent to

(49) 2c3+4cc˙+c¨=0,ddtln⁡ρt,1=−c.

Note that (49)_1_ is a special case of equation (7) in [10] when we set the parameter *N* in (7) to be zero. Thus, by Theorem  2.1 in [10], the results (1) and (2) in the proposition follow.

**Remark 12 .**

From (49)_2_ and (15), the density function *ρ* is given by

(50) $$\begin{array}{c} \rho \left(\;t,r\;\right)=\rho \left(\;0,1\;\right)e^{-\int_{0}^{t}c\left(s\right)ds}e^{-\left(\left(c^{2}+\dot{c}\right)/K\right)\left(\left(1/2\right)r^{2}-r+1/2\right)}. \end{array}$$

Thus, the total mass is finite if $c^{2}(0)+\dot{c}(0)>0$ and is infinite if $c^{2}(0)+\dot{c}(0)<0$. From (1) and (2) in the proposition, we see that blowup can occur only when the total mass is infinite.

**Proof of Theorem 1.**

Theorem 1 is followed from Propositions 8–11.

Finally, we are ready to present the proof of Corollary 2.

**Proof of Corollary 2.**

Let $c=\dot{a}/a$ in (49)_1_. One has

(51) $$\begin{array}{c} \frac{\dddot{a}}{a}+\frac{\dot{a}\ddot{a}}{a^{2}}=0. \end{array}$$

It follows that

(52) ddtaa¨=0,aa¨=λ,

where $\lambda ≔a_{0}\ddot{a}(0)$. Thus, *a* satisfies (6). Consider (6) with initial conditions

(53) a0=a0>0,a1≔a˙0.

Set

(54) $$\begin{array}{c} T^{*}≔\sup\left\{\;t\geq 0:a>0\;\;on\;\;\left[\;0,t\;\right)\;\right\}>0. \end{array}$$

First, note that if *T* ^*∗*^ is finite, then the one-sided limit lim_*t*→*T*^*∗*^_ *a*(*t*) must be zero. More precisely, if *T* ^*∗*^ is finite and lim_*t*→*T*^*∗*^_ *a*(*t*) > 0, then we can extend the solution by solving (6) with initial condition *a*(*T* ^*∗*^)≔lim_*t*→*T*^*∗*^_ *a*(*t*) > 0. This contradicts the definition of *T* ^*∗*^.

Next, suppose *λ* < 0, and then
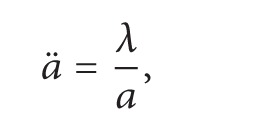
(55)

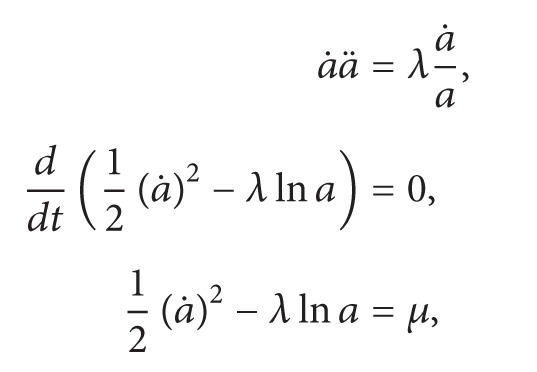
(56)where *μ*≔(1/2)*a*_1_^2^ − *λ*ln⁡*a*_0_. It follows that

(57) μ+λln⁡a=12a˙2≥0,ln⁡a≤−μλ,a≤e−μ/λ.

From (55), one has

(58) a¨≤λeμ/λ,at≤12λeμ/λt2+a1t+a0.

As the coefficient of *t* ^2^ is negative, we see that *a*(*t*) will be negative if *t* is sufficiently large. This implies that $T^{*}<\left(a_{1}+\sqrt{a_{1}^{2}-2a_{0}\lambda e^{\mu /\lambda }}\right)/-\lambda e^{\mu /\lambda }$ is finite.

On the other hand, from (55), one has

(59) a˙=a1+∫0tλasds≤a1+λeμ/λt,limt→T∗⁡a˙≤a1+λeμ/λT∗<−a12−2a0λeμ/λ<0.

Thus,

(60) $$\begin{array}{c} \underset{t\to T^{*}}{\lim}\frac{\dot{a}}{a}=-\infty . \end{array}$$

Thus, *u* → −*∞* and *ρ* → *∞* as *t* → *T* ^*∗*^.

For *λ* > 0, one has

(i) *a* ≥ *e* ^−*μ*/*λ*^ > 0,
(ii) *T* ^*∗*^ = *∞*,
(iii) (61) $$\begin{array}{c} \rho \left(\;t,r\;\right)=\frac{a_{0}\rho \left(0,1\right)}{a\left(t\right)}e^{-\left(\lambda /2Ka^{2}\left(t\right)\right){\left(r-1\right)}^{2}}. \end{array}$$

From (i), (ii), and (iii) above, we see that *ρ* is bounded above by

(62) $$\begin{array}{c} \rho \left(\;0,1\;\right)a_{0}e^{\mu /\lambda }. \end{array}$$

Moreover, we have lim_*t*→*∞*_ *a*(*t*) = *∞*. This is because if lim_*t*→*∞*_ *a*(*t*) is finite, then *a*(*t*) is bounded by some positive number *M* > 0. But, from (6), one has

(63) a¨≥λM,at≥λ2Mt2+ta1+a0,

which implies that *a* is unbounded as the coefficient of *t* ^2^ is positive.

Next, we show that

(64) $$\begin{array}{c} \underset{t\to \infty }{\lim}\frac{\dot{a}}{a}=0. \end{array}$$

If ${lim}_{t\to \infty }\dot{a}$ is finite, then (64) is clearly held. If ${lim}_{t\to \infty }\dot{a}$ is not finite, then

(65) $$\begin{array}{c} \underset{t\to \infty }{\lim}\frac{\dot{a}}{a}=\underset{t\to \infty }{\lim}\frac{\ddot{a}}{\dot{a}}=\underset{t\to \infty }{\lim}\frac{\lambda }{a\dot{a}}=0. \end{array}$$

Thus, for *λ* > 0, (*ρ*, *u*)→(0,0) as *t* → *∞*.

As the cases for *λ* = 0 can be verified trivially, the proof is complete.

## Figures and Tables

**Table 1:** 

*α*	1/3	1/2	2/3	2

3*α* − 1 (*D*_1_)	0	1/2	1	5

2*α* (*D*_2_)	2/3	1	4/3	4

2*α* − 1 (*D*_3_)	−1/3	0	1/3	3

*α* + 1 (*D*_4_)	4/3	3/2	5/3	3

*α* (*D*_5_)	1/3	1/2	2/3	2

*α* − 1 (*D*_6_)	−2/3	−1/2	−1/3	1

1 (*D*_7_)	1	1	1	1

0 (*D*_8_)	0	0	0	0

−1 (*D*_9_)	−1	−1	−1	−1
